# Imaging mass spectrometry analysis of ubiquinol localization in the mouse brain following short-term administration

**DOI:** 10.1038/s41598-017-13257-8

**Published:** 2017-10-11

**Authors:** Yukina Tatsuta, Kazuaki Kasai, Chitose Maruyama, Yoshimitsu Hamano, Kazuhiko Matsuo, Hajime Katano, Shu Taira

**Affiliations:** 1 Department of Bioscience, Fukui Prefectural University, Eiheiji, Fukui, 910-1195 Japan; 2 Takasago Analysis Center, Kaneka Techno Research Corporation, 1-8 Miyamaecho Takasagocho, Takasago, Hyogo, 676-8688 Japan

## Abstract

We analyzed the localization of ubiquinol, the reduced form of coenzyme Q10 (Re-CoQ10), in mouse brain sections using matrix-assisted laser desorption/ionization Fourier transform ion cyclotron resonance imaging mass spectrometry (IMS) to evaluate the effect of dietary Re-CoQ10 in mouse brain. Mice were orally administered Re-CoQ10 for 14 days and brain Re-CoQ10 content was subsequently quantified using liquid chromatography-mass spectrometry. IMS was employed to visualize Re-CoQ10 at a resolution of 150 μm in the mouse brain. Increased Re-CoQ10 was observed in the corpus callosum, hippocampus, midbrain, cerebellum, brain stem, substantia nigra and striatum. These regions are related to movement, memory and vital life functions. Thus, we demonstrated the effect of Re-CoQ10 administration on the specific localization of Re-CoQ10 in the brain.

## Introduction

**Figure 1:**
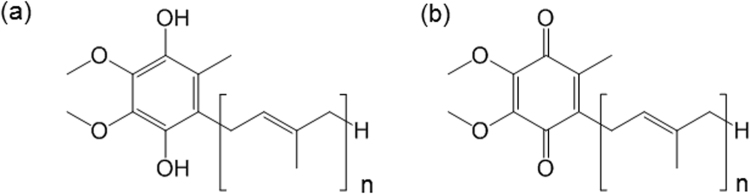
Chemical structures of ubiquinol (**a**) and the oxidized form of coenzyme Q10 (n = 10) and coenzyme Q9 (n = 9) (**b**).

Coenzyme Q10 (CoQ10) is widely known as a dietary supplement that promotes health functions as well as clinical benefits for cardiac and neurodegenerative diseases. CoQ10 exists in two forms, oxidized (ubiquinone-10; Ox-CoQ10) and reduced (ubiquinol-10; Re-CoQ10) (Fig. 1). In the human body, Re-CoQ10 has an essential role in the production of adenosine triphosphate (ATP) in the electron transport system of the mitochondrial respiratory chain. Moreover, Re-CoQ10 is a powerful lipid-soluble antioxidant that plays an intrinsic role in protecting circulating lipoproteins against oxidative damage and mitochondria and other cellular components from reactive oxygen species generated by mitochondrial respiration^1^. CoQ10 levels are known to decrease with aging in various mammalian species including humans, as well as in those who are under strong oxidative stress^2^. Because of this loss and its essential biological roles, several commercial CoQ10 products are sold as dietary supplements. Moreover, many recent studies reported that CoQ10 exerts neuroprotective effects and improved the phenotype of certain brain diseases, including Alzheimer’s disease, amyotrophic lateral sclerosis and Huntington’s disease^3–5^. If oral ingestion of Re-CoQ10 affects to the amount and localization of CoQs in brain, Re-CoQ10 has the potential as easy improvement method for not only health care but also cranial nerve disease. However, to our knowledge, no reports have demonstrated the detailed accumulation of CoQ10 in various brain regions. Detailed information regarding CoQ10 localization can be used to elucidate the mechanism of neuroprotective effects in the brain. Conventional staining methods require fluorescence or antibodies to visually reveal the localization of target molecules, whereas the use of two-dimensional mass spectrometry (MS) analysis of biomedical tissues, also known as imaging mass spectrometry (IMS), has become more commonplace for analyzing the distribution of analytes in several fields, including biology^6–8^, drug development^9^, pharmacology^10^, food science^11,12^, agriculture^13–15^ and brain science^16^. It is worth noting that distinguishing spatial information of CoQs and their visualization are difficult when using techniques based on chromatography. However, IMS can easily recognize and provide such spatial information. Following two-dimensional MS measurements on sample sections at regular intervals, reconstruction of target signals is obtained as an ion image. Thus, IMS enables simultaneous detection of multiple analytes even in the absence of target-specific markers, such as antibodies^6,8,10^. Furthermore, IMS has the potential to identify new biomarkers as well as important common target molecules.

Here, we adopted three techniques in an effort to reveal the localization of Re-CoQ10 in the mouse brain. First, to detect multiple molecules in raw tissue sections, matrix-assisted laser desorption/ionization (MALDI) was used as a soft ionization technique, wherein a chemical matrix is used to assist ionization of the molecules of interest^17,18^. Second, to classify the molecular species from their exact mass without tandem mass spectrometry, Fourier-transform ion cyclotron resonance (FT-ICR) mass spectrometry was used, which offers high resolution (R = 10^5^–10^6^) and accuracy (Δ*m* = 10^−4^–10^−3^)^19–21^. Lastly, CoQs were visualized at a resolution of 150 μm in the mouse brain by IMS. We mainly used IMS to investigate ubiquinol localization in the mouse brain following a 14-day oral administration period. To our knowledge, this is the first report to visually determine the localization of CoQs in regions associated with memory and behavior, and compare the increase of ubiquinol in the mouse brain after continuous oral administration of ubiquinol.

## Results and Discussion

### Liquid chromatography-mass spectrometry (LC- MS) for quantitative analysis of Coenzyme Qs in mouse brain

**Figure 2:**
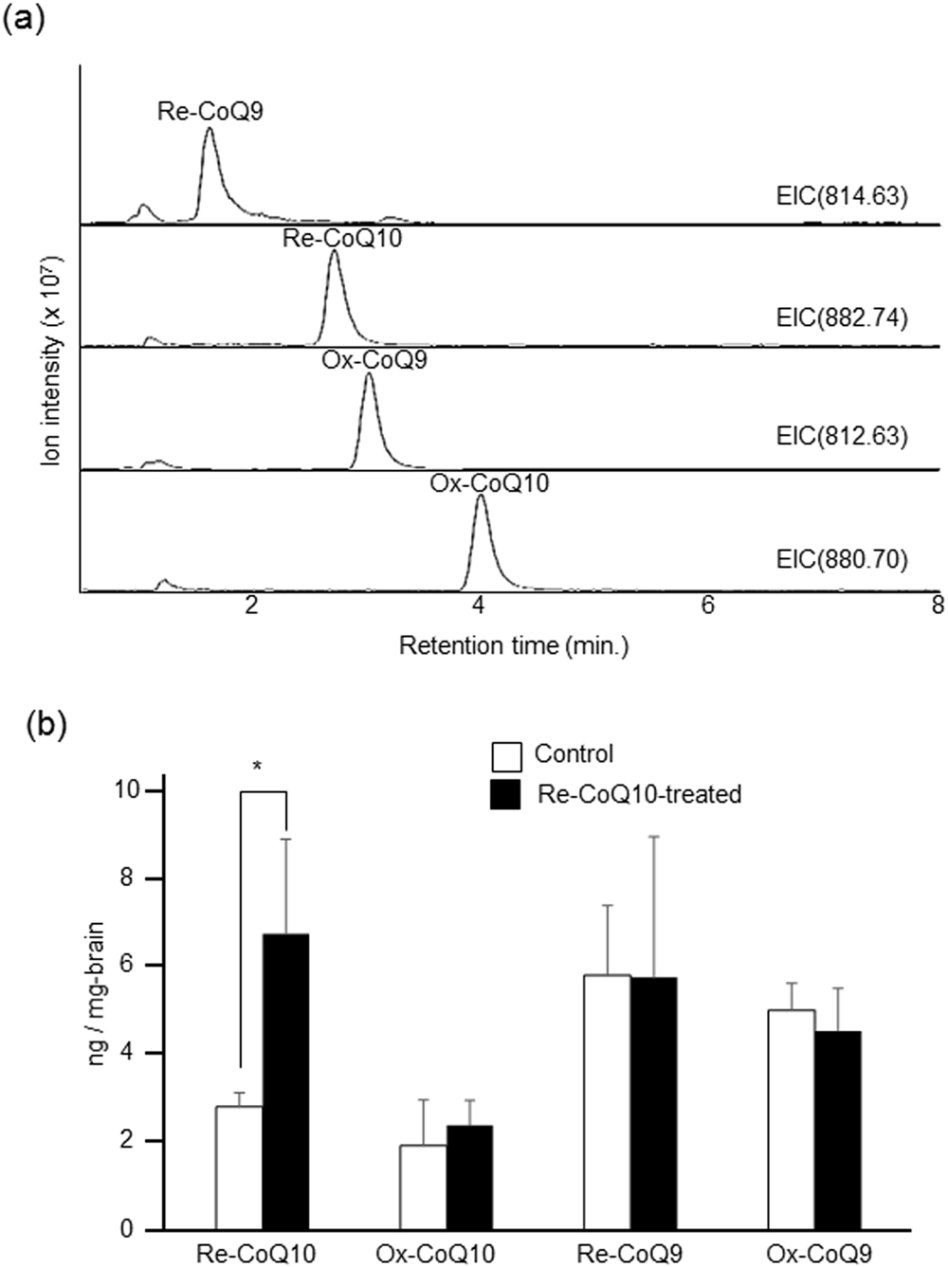
Extracted ion chromatography analysis of Re-CoQ10-treated mice (**a**). Quantitative comparison of the amount of CoQs in Re-CoQ10-treated and control mice (**b**). Obtained values were calculated per unit area. The values are expressed as mean + SEM. *p < 0.05 with student’s t-test. n = 8.

All CoQ standards were separated under the same gradient conditions. We selected four different ions that correspond to Re-CoQ9 [*m/z* 814.63; retention time (RT): 1.5 min], Re-CoQ10 (*m/z* 882.74; RT: 2.7 min), Ox-CoQ9 (*m/z* 812.63; RT: 3.2 min) and Ox-CoQ10 (*m/z* 880.70; RT: 4.3 min). These retention times are reasonable for reversed-phase LC because the method is dependent on molecular hydrophobicity, which is affected by the chain length and redox state of ubiquinone. A representative selected ion chromatograph of CoQs is shown in Fig. 2a. CoQs were quantified using a standard method based on peak area. The concentrations of Re-CoQ9, Re-CoQ10, Ox-CoQ9 and Ox-CoQ10 were 5.9 ± 1.6, 2.8 ± 0.3, 5.0 ± 0.6 and 1.9 ± 1.0 ng/mg brain in control mice and 5.8 ± 3.0, 6.8 ± 2.1, 4.5 ± 0.9 and 2.4 ± 0.5 ng/mg brain in Re-CoQ10-treated mice, respectively (Fig. 2b). Significantly elevated levels of Re-CoQ10 were observed in Re-CoQ10-treated mice compared to control mice (P < 0.05). No differences were observed for the other CoQs, although Re-CoQ9 tended to be increased in Re-CoQ10-treated mice. This result indicates that Re-Co10 administration directly affects Re-CoQ10 concentration in the brain.

### Two-dimensional mapping of coenzyme Qs by IMS

Using the ubiquinol standard, we confirmed the protonated ubiquinol signal (*m/z* 865.704). In the representative mass spectrum obtained for the tissue section sprayed with matrix solution, we detected various high-resolution signals in an *m/z* range of 800–900 using MALDI-TOF MS. As a result, we confirmed ionization of the ubiquinol standard. Moreover, we performed component analysis by MS/MS. We obtained a benzoquinone signal that fragmented from the precursor ion of ubiquinol. These results indicate that ubiquinol could be detected by MS using tissue sections.

**Figure 3:**
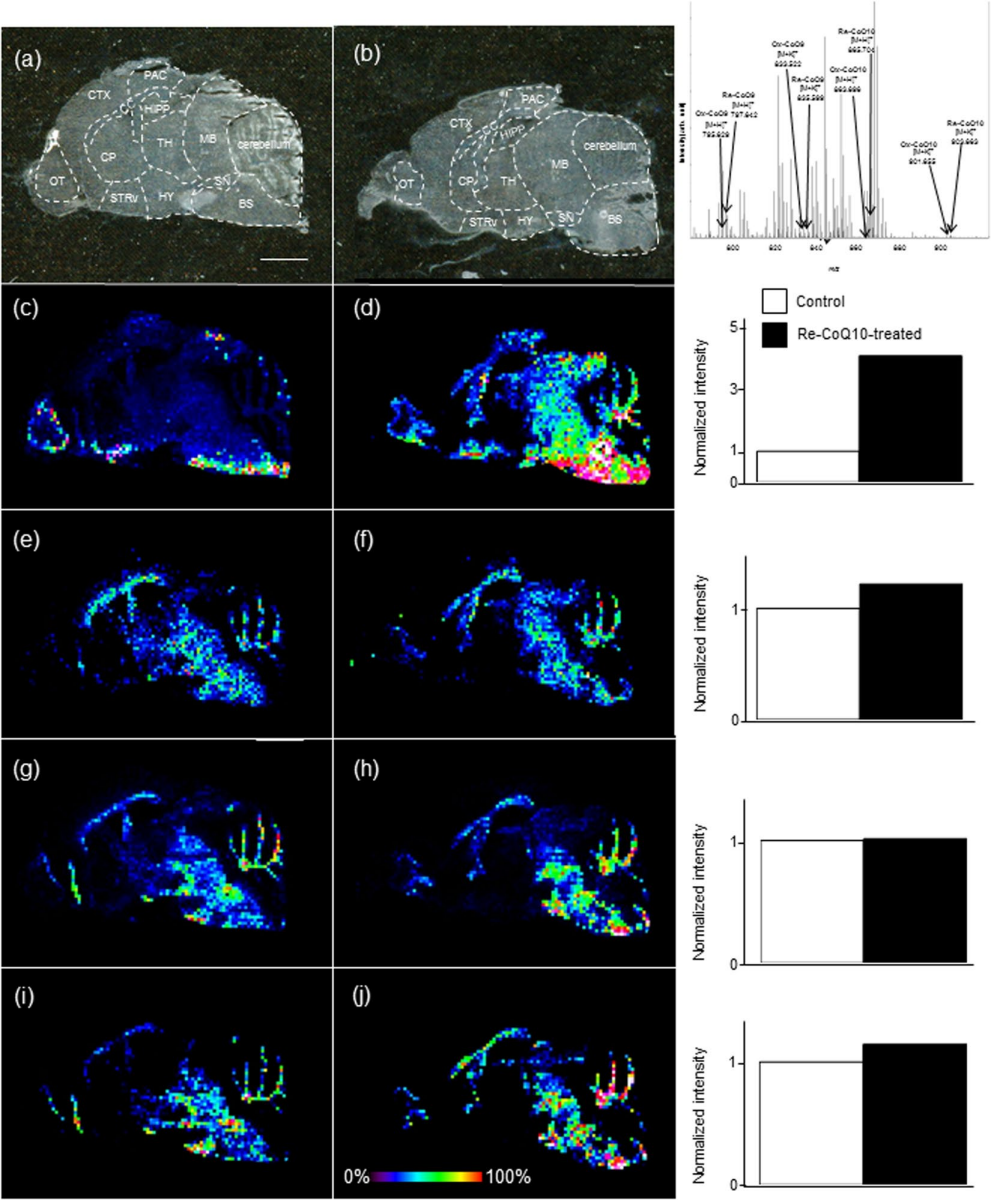
Imaging mass spectrometry (IMS) of CoQs. Optical image of a sagittal tissue section from the mouse brain (**a**) control and (**b**) Re-CoQ10-treated mouse). MS spectra reconstructed image of Re-CoQ10 (**c**) control and (**d**) Re-CoQ10-treated mouse, Ox-CoQ10 (**e**) control and (**f**) Re-CoQ10-treated mouse Re-CoQ9 (**g**) control and (**h**) Re-CoQ10-treated mouse and Ox-CoQ9 (**i**) control and (**j**) Re-CoQ10-treated mouse. The obtained image data is presented using a rainbow scale and normalized versus total ion count (right bar chart); scale bars, 500 μm. Olfactory tuberculum, OT; caudate-putamen, CP; corpus callosum, CC; parietal association cortex, PAC; midbrain, MB; thalamus, TH; hippocampus, HIPP; hypothalamus; HY, substantia nigra, SN; ventral striatum, STRv; brain stem (BS), cerebral cortex (CTX).

Next, we performed *in situ* localization analysis of sagittal mouse brain sections using IMS. The signal correlated target CoQs (protonated and potassium adducted ions) were confirmed in biological tissues (top right panel, Fig. 3). Optical images of brain sections used for IMS were obtained (Fig. 3a,b). To establish the relationship between selected CoQ ions and the MS image, we used merged images of protonated and potassium adducted ions to reveal the localization of total CoQs. Re-CoQ10 (*m/z* 865.704 as protonated ion and 903.663 as potassium adducted ion) localization in Re-CoQ10-treated and control mice was similar in the corpus callosum (CC), parietal association cortex (PAC), hippocampus (HIPP), midbrain (MB), cerebellum, brain stem (BS), substantia nigra (SN), thalamus (TH), hypothalamus (HY),ventral striatum (STRv), caudate-putamen (CP), and olfactory tuberculum (OT), respectively. Conversely, Re-CoQ10 was largely absent in the cerebral cortex (CTX). Normalized ion intensity (right-most panels, Fig. 3) in whole brain sections of Re-CoQ10-treated mice increased 4-fold compared to that in control mice (Fig. 3c,d). Ox-CoQ10 (*m/z* 863.696 and 901.655) localized to the CC, MB, SN, BS and cerebellum. However, localization and the relative ratio of Ox-CoQ10 did not differ substantially between Re-CoQ10-treated and control mice (Fig. 3e,f). Moreover, Ox-CoQ10 was largely absent in the OT, CP and striatum of the frontal cortex. Both Re-CoQ9 (*m/z* 797.642 and 835.599) and Ox-CoQ9 (*m/z* 795.629 and 833.522) were observed in the OT, CP, CC, MB, SN, BS and cerebellum. Normalized intensities in the whole brain were similar between Re-CoQ10-treated and control mice (Fig. 3g–j). The relative ion intensity of CoQs was in good agreement with the LC-MS results.

**Figure 4:**
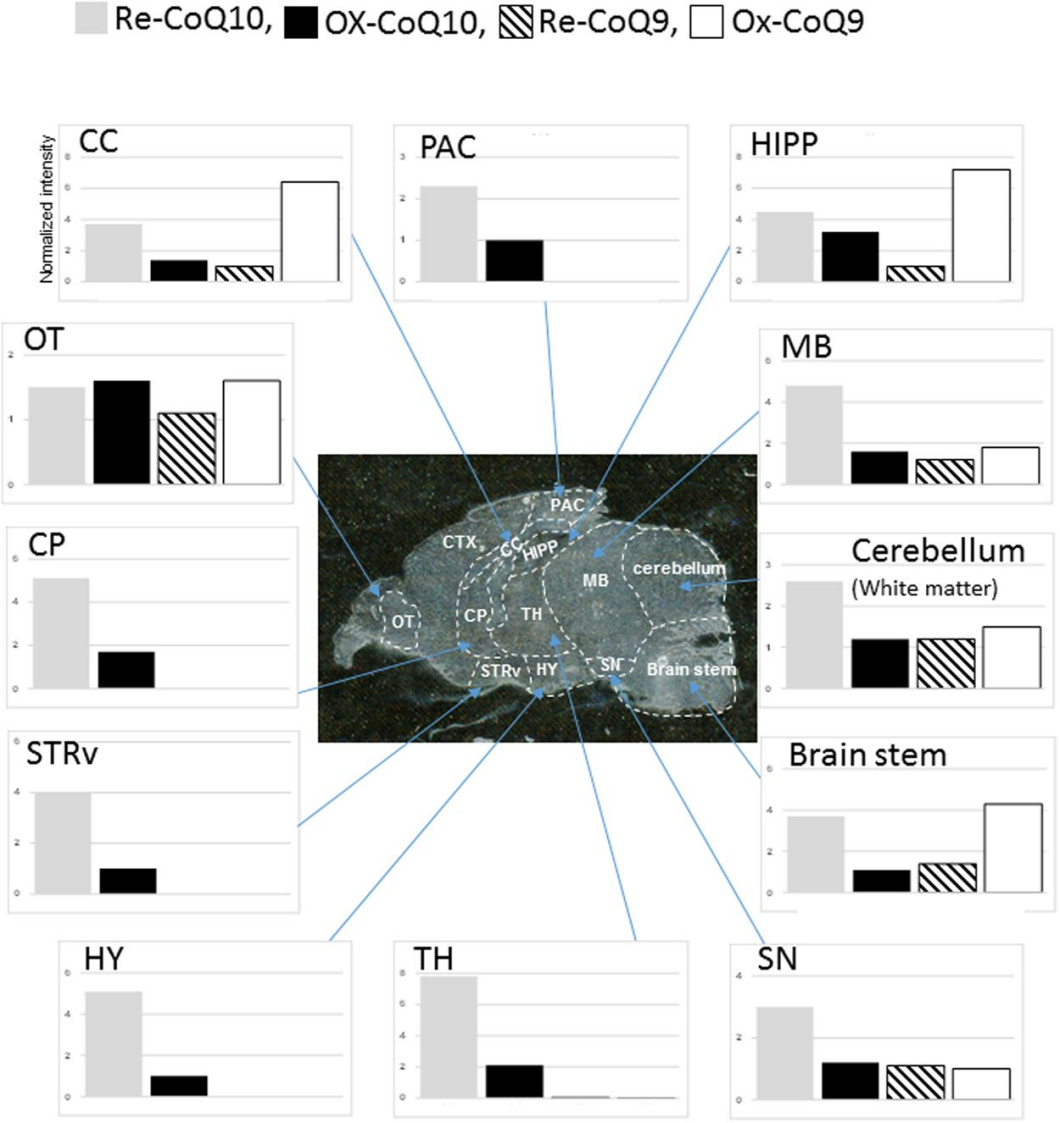
Regional distribution of CoQs in Re-CoQ10-treated mice. The data was normalized to a control mouse.

A regional analysis was subsequently performed by normalization of MS intensities of the four CoQs in Re-CoQ10-treated mice to that of control mice (Fig. 4). Re-CoQ10 concentrations were increased by about 4-fold in the STRv and CP, areas associated with motility function. Particularly, STRv and CP receive monoamines such as dopamine, norepinephrine and 3,4-dihydroxyphenylacetic acid, which are produced in the SN and associated with consciousness^16^. Similarly, the Re-CoQ10 concentration was increased by 3-fold in SN in Re-CoQ10-treated mice. The HIPP, which is involved in short-term memory, exhibited a 4-fold increase of Re-CoQ10 and Ox-CoQ9 in Re-CoQ10-treated mice compared to control mice. Thus, we considered Re-CoQ10 is involved in consciousness and memory system. In the cerebellum, which is associated with modulation of movement, the Re-CoQ10 concentration was increased by about 2.5-fold in Re-CoQ10-treated mice compared to control mice, although concentrations of other CoQs were similar between groups. Interestingly, Re-CoQ10 was mainly localized in white matter in the cerebellum. This area is abundant in neuraxons, suggesting that Re-CoQ10 may accumulate in nerve-dense areas such as the central nervous system due to high mitochondrial activity^22,23^. Furthermore, increased Re-CoQ10 levels of 2–3-fold in Re-CoQ10-treated mice were observed in the CC and PAC, which receive and process information. Moreover, Re-CoQ10 concentrations were increased about 4-fold in the BS in Re-CoQ10-treated mice, which is responsible for the maintenance of life functions, and the MB, which is associated with sense and temperature regulation. However, all CoQs were hardly detected in the CTX. A previous report showed that CoQ10 increased in the CTX following a 60-day administration period^24^. The discrepancy between their results and our results is attributable to the short administration period used in the present study. For sensory regions HY, TH and OT, Re-CoQ10 concentrations increased about 4–8-fold in Re-CoQ10-treated mice compared to control mice in the HY and TH. However, Re-CoQ10 levels in the OT were similar between groups. The mouse OT directly receives smell information and is involving in searching for food and escaping from danger. Therefore, Re-CoQ10 was predominantly consumed and rapidly recycled in control mice, resulting in preferential distribution of Re-CoQ10 in basal systems such as the OT. Conversely, oral administration of Re-CoQ10 resulted in progressive increases in Re-CoQ10 in brain regions associated with the maintenance of life as well as movement, information processing and memory. This finding suggested that abundant supply of Re-CoQ10 resulted in Re-CoQ10 distribution to significant areas requiring energy while remaining at the usual site (OT).

Excess decreased Re-CoQ10 concentrations in the brain are considered an indicator of health disturbance^3^. Typically, Brain specimens from regions correlated with the diseases were investigated for redox balance of CoQs by chromatographic technic. However, in this study, we demonstrated IMS could easily and visually distinguish the redox balance of CoQs. Increased Re-CoQ10 concentrations were observed in the HIPP, SN, STRv and CP, which are damaged by major central nervous system (CNS) diseases such as Alzheimer’s^25^, Parkinson’s^5^ and Huntington’s^26^ disease. Thus, visualization and discrimination of CoQs may be a potential marker of CNS diseases as well as myocardial infarction, diabetes and cancer^27^. However, because we only investigated the distribution of CoQs in young mice, similar studies should be performed using older animals to elucidate the potential involvement of CoQ in brain diseases. Furthermore, due to holistic observation, IMS provided unexpected regional information in addition to information of target regions. To our knowledge, this is the first report to suggest a difference in Re-CoQ10 concentrations in the BS, which controls life support.

Regarding Re-CoQ10 absorption, update of administered Re-CoQ10 appears to be similar to that of fat-soluble vitamins and nutrients. Re-CoQ10 is absorbed in the small intestine without affecting gastric digestion^28^ and transported to organs via the lymphatic system^29^. However, the blood-brain barrier (BBB) presents a problem for the delivery of therapeutics to the brain, as large molecules cannot pass through the BBB. The BBB exists throughout the brain and is formed by tight junctions between endothelial cells of the capillary vessels. Previous reports described that CoQ10 is able to cross the BBB^4,24^, which was supported by our results. In addition, many regions in the brain, such as circumventricular organs, organum vasculosum laminae terminalis, hypothalamus, epiphysis and posterior pituitary, are permeable to various materials in a manner similar to peripheral vessels. In this context, we propose that periodic administration of Re-CoQ10 affects CoQs concentrations in the brain. Re-CoQ10 may induce mitochondrial energy production, leading to enhanced cellular activity at Re-CoQ10-localized regions. This finding suggests Re-CoQ10 can be used as a potential marker of mitochondrial activity as an alternative to ATP, as evaluation or imaging of ATP is difficult and labor-intensive due to its short half-life^30,31^. Moreover, as Re-CoQ10 localization was readily visualized by IMS, we anticipate this technique will facilitate investigation of brain activity in fields of healthcare, neurochemistry and pathology.

## Methods

### Chemicals

Re-CoQ10, Ox-CoQ10, Re-CoQ9 and Ox-CoQ9 were obtained from Kaneka Corporation (Osaka, Japan). Acetonitrile, 2-propanol, carboxymethyl cellulose (CMC), ethanol, xylene, hematoxylin and eosin were obtained from Wako Pure Chemical Industries, Ltd. (Tokyo, Japan). α-Cyano-4-hydroxycinnamic acid (CHCA) was purchased from Nacalai Tesque (Tokyo, Japan). All other chemicals were obtained from either Wako or Sigma-Aldrich (St. Louis, MO). All reagents were high-performance liquid chromatography (HPLC) grade.

### Mouse model of ubiquinol administration

Female ICR mice (n = 8) (12-week-old; body weight, 30–33 g; Clea Japan, Tokyo, Japan) were used in this study in accordance with the institutional Animal Experimental Guidelines of Fukui Prefectural University and approved by the Laboratory Animal Care and Use Committee of Fukui Prefectural University (Permission No. 17-3). Mice were fed laboratory chow and allowed free access to water. For the oral administration experiment, we used a feeding needle to administer ubiquinol (300 mg/kg) containing corn oil continuously for 14 days to female ICR mice (n = 4). Mice fed a normal saline solution were used as the control group (n = 4).

### Preparation of tissue sections for MALDI-IMS

All mice were anesthetized with 4% isoflurane by an anesthetic instrument (SN-487-OT, Natsume SEISAKUSHO Co. Ltd., Tokyo, Japan). Blood perfusion by normal saline/heparin (1 unit/mL) solution was achieved to remove blood. The brains were dissected by surgical scissors and tweezers at room temperature, embedded into a super cryo-embedding medium (Section Lab Co. Ltd., Hiroshima, Japan), flash-frozen in liquid N_2_ and stored at −80 °C until use. The left hemisphere was embedded in 2% CMC and cut into serial sagittal sections (50 μm) using a cryostat (CM-3050 S; Leica Microsystems, Wetzlar, Germany) at −25 °C and thaw-mounted on indium tin oxide (ITO)-coated slides for IMS. The right hemisphere was used for quantitative analysis by liquid chromatography MS (LC-MS). After thaw-mounting, ITO slides were allowed to dry in a desiccator until matrix coating. Unconsciousness induced by anesthesia was confirmed before blood collection and animals were humanely euthanized before brain extraction according to the American Veterinary Medical Association Recommendations (AVMA Guidelines for the Euthanasia of Animals: 2013 Edition).

### Detection of targets by MALDI-TOF MS

Ubiquinol (10 pmol/μL) was dissolved in isopropanol and a suspension containing ubiquinol and CHCA (10 mg/mL) was placed on a target plate using a pipette. Ionization of standard ubiquinol was confirmed by MALDI-TOF MS (ultrafleXtreme, Bruker Daltonik GmbH, Mannheim, Germany). The analyte surface was irradiated with 1,000 laser shots and TOF spectra were acquired in positive ion mode.

### Quantitative analysis of CoQs by LC-MS

ESI-MS was used to quantitate the ubiquinol concentration. The mouse right hemisphere was homogenized in isopropanol using a homogenizer and the obtained suspension was centrifuged to remove insoluble materials. The supernatant was then analyzed by LC-MS. LC was performed with an HPLC system (Infinity 1260, Agilent Technologies, Santa Clara, CA) consisting of a binary pump, degasser, autosampler and thermostat-controlled column oven. An ODS column (YMC-PAC ODS-A, 100 × 20 mm, i.d., 3 μm, YMC Co., Ltd., Kyoto, Japan) was used. The mobile phase of methanol-ethanol (35:65, v/v) was employed at a flow rate of 0.4 mL/min. The quadrupole TOF mass spectrometer (maXis, Bruker Daltonik GmbH Mannheim, Germany) was used and TOF spectra were acquired in positive ion mode.

### Matrix coating on brain section and MALDI-FT-ICR IMS

Brain sections were manually sprayed with matrix (CHCA-acetonitrile/2-propanol/water = 50/49/1) using an artistic airbrush (Procon Boy FWA Platinum 0.2-mm caliber airbrush, Mr. Hobby, Tokyo, Japan). We initially maintained a distance of 20 cm between the airbrush and target ITO glass during matrix coating to form the matrix seed and then changed the distance between the airbrush and target ITO glass to 25 cm during matrix coating to extract the target molecule on the section and form the matrix crystal. MALDI mass spectra were acquired on MALDI-FT-ICR (solariX, Bruker Daltonik GmbH) equipped with a Nd:YAG laser. To detect the laser spot area, sections were scanned and laser spot areas (1,000 shots) were detected with a spot-to-spot center distance of 150 μm in each direction of the brain. Signals between *m/z* 500–1,000 were corrected. The section surface was irradiated with YAG laser shots in positive ion mode. The laser power was optimized to minimized in-source decay of CoQs. Obtained MS spectra were reconstructed into MS images with a mass bin width of *m/z* ± 0.001 from the exact mass using FlexImaging 4.0 software (Bruker Daltonik GmbH). IMS data are depicted using a rainbow scale over a single range on a linear scale. The red pixel represents the highest signal intensity (100%) of the particular ion, and the black pixel represents the lowest signal (0%). The peak intensity value of the spectra was normalized by dividing them by the total ion current to achieve semi-quantitative analysis between Re-CoQ10-treated and control mice. Optical images of brain sections were obtained by a scanner (GT-X820, Canon, Tokyo, Japan), followed by MALDI-FT-ICR IMS of the sections.

### Statistical analysis

Measurements are reported as mean ± SEM. We performed unpaired two-tailed Student’s t-test for single comparisons. Significance was considered at P < 0.05. All analyses were performed by Excel 2013 software (Microsoft, Redmond, WA).
